# Stream biodiversity and function benefit from forest fragments in agricultural landscapes

**DOI:** 10.1007/s00442-026-05977-4

**Published:** 2026-09-24

**Authors:** Paula M. de Omena, Larissa C. Costa, Paula C. R. Oliveira, Rebecca Oester, Andreas Bruder, Marcelo S. Moretti

**Affiliations:** 1 Laboratory of Aquatic Insect Ecology, University of Vila Velha, Av. Comissário José Dantas de Melo 21, 29102-920 Vila Velha, ES Brazil; 2 Graduate Program in Plant Biotechnology, University of Vila Velha, Av. Comissário José Dantas de Melo 21, 29102-920 Vila Velha, ES Brazil; 3 College of Agriculture, University of São Paulo (ESALQ), Av. Pádua Dias 11, 13418-900 Piracicaba, SP Brazil; 4 Institute of Microbiology, University of Applied Sciences and Arts of Southern Switzerland, via Flora Ruchat Roncati 15, CH-6850 Mendrisio, Switzerland; 5 Department of Evolutionary Biology and Environmental Studies, University of Zurich, Winterthurerstr. 190, CH-8057 Zurich, Switzerland; 6 Department of Aquatic Ecology, Swiss Federal Institute of Aquatic Science and Technology, Überlandstrasse 133, CH-8600 Dübendorf, Switzerland

**Keywords:** Agricultural catchments, Aquatic invertebrates, Leaf breakdown, Riparian vegetation, Atlantic Forest streams

## Abstract

Riparian forests buffer stream ecosystems from intensive land use. However, with ongoing land-use changes, riparian forests often remain as isolated fragments. Despite this, the relevance of isolated forest fragments in deforested landscapes remains unclear. We evaluated how downstream forest fragments can buffer the effects of upstream deforestation and whether upstream forest fragments can mitigate the impacts of downstream deforestation on abiotic conditions, biodiversity, and stream functioning. Additionally, we assessed how subsidized leaf litter of varied quality and diversity influences leaf-associated invertebrate assemblages and leaf litter breakdown rates. We conducted leaf breakdown experiments in eight streams within agricultural catchments—four flowing from an upstream forest fragment to a deforested reach, and four from a deforested area to a downstream forest fragment. Downstream forest fragments reduced the effects of upstream deforestation, as evidenced by higher shredder diversity and lower scraper abundance in these reaches than in the upstream deforested reaches. Upstream forest fragments also mitigated downstream deforestation by reducing sediment load, moderating water temperatures, and enhancing substrate diversity. The taxonomic similarity between the forest upstream and the deforested downstream reaches highlighted the role of forest fragments as sources of invertebrates and organic matter for deforested reaches. In forest upstream reaches, invertebrates increased the breakdown rates of C-labile fast-decomposing leaf species, whereas leaf diversity accelerated the processing of nitrogen-fixing slow-decomposing leaves. These results demonstrate that the presence of forest fragments in agricultural catchments benefits water quality, invertebrate communities, and leaf litter breakdown, thereby contributing to the resilience of stream ecosystems to land-use changes.

**Supplementary Information:**

The online version contains supplementary material available at https://doi.org/10.1007/s00442-026-05977-4.

## Introduction

Forest stream ecosystems and their aquatic communities depend intimately on the conditions of the riparian zone (Tolkkinen et al. 2020). Riparian forests are crucial in mitigating human impacts such as land-use change by acting as natural filters that capture sediments (Dunn et al. 2022), nutrients (Mayer et al. 2007), and pollutants (Aguiar et al. 2015) from surface runoff. They also regulate the water temperature (Kail et al. 2021) and stabilize stream banks (Krzeminska et al. 2019). Moreover, the inputs of organic matter, primarily in the form of leaf litter from multiple tree species, are crucial for maintaining biodiversity by increasing habitat heterogeneity and providing food resources and shelter for aquatic biota (Deere et al. 2022; Marques et al. 2021). Despite their importance in preserving biodiversity and ecosystem function, riparian forests are frequently suppressed for agricultural expansion and human settlement (Michalski et al. 2010; Rezende et al. 2018). In some catchments, many of the remaining riparian forests only exist in isolated fragments within a mosaic of altered landscapes (Bennett and Saunders 2011; Fischer and Lindenmayer 2007). While the importance of riparian forests as buffers is widely acknowledged, further research is needed to understand the contribution of forest fragments to reducing the impacts of deforestation on stream ecosystems.

The deforestation of riparian zones disrupts aquatic‒terrestrial linkages, affecting the supply of allochthonous resources to aquatic food webs (England and Rosemond 2004). It also triggers changes in water parameters, such as pH, oxygen concentration, and streambed structure, thereby diminishing ecosystem integrity (Machado-Silva et al. 2022; Taniwaki et al. 2017). These changes also frequently impair aquatic diversity due to a reduction in the abundance of species that are sensitive to disturbance, such as invertebrate taxa belonging to the orders Ephemeroptera, Plecoptera, and Trichoptera (EPT) (Oester et al. 2023; Petsch et al. 2021; Silva-Araújo et al. 2020). Many of these taxa belong to the functional feeding group of shredders, which consume mainly allochthonous resources such as leaf litter (Tachet et al. 2010). Riparian deforestation can thus increase the abundance of invertebrates that rely on in-stream primary productivity (i.e., scrapers) and tolerant taxa such as chironomids and oligochaetes (Espinoza-Toledo et al. 2021; Iñiguez-Armijos et al. 2018). Riparian deforestation often causes reduced leaf breakdown rates (Oester et al. 2023; Silva-Araújo et al. 2020) and increased primary production (e.g., of macrophytes and periphyton) (Gücker et al. 2009; Machado-Silva et al. 2022). Therefore, such streams may also show a shift in ecosystem functioning and in the dominance of energy flow paths from allochthonous to autochthonous (Bleich et al. 2015).

Streams are substantially influenced not only by adjacent terrestrial ecosystems but also by upstream conditions in the stream catchment (Harding et al. 2006; Vannote et al. 1980). Therefore, the potential of forest fragments to mitigate human impacts in streams may depend on their location, i.e., upstream or downstream of a deforested reach. In agricultural‒forest transitions, forest fragments have been suggested to alleviate the adverse effects of upstream deforestation in stream ecosystems (Goss et al. 2014; Suga and Tanaka 2013). However, the effectiveness of these fragments varies depending on the specific ecological attribute being assessed, with findings often inconsistent across studies (Goss et al. 2014). For example, while some studies have documented an increased richness of EPT in deforested streams upon entering forest fragments (Neill et al. 2001; Scarsbrook and Halliday 1999), others have reported no change or even a slight decrease in EPT richness (Harding et al. 2006; Suga and Tanaka 2013). On the other hand, forest fragments located deforested upstream reaches may act as sources of organic matter, diverse leaf litter, and aquatic organisms (Lancaster et al. 1996; Wipfli et al. 2007), contributing to habitat heterogeneity and sustaining a greater diversity of invertebrates across functional feeding groups (Lima et al. 2022). Differences among leaf litter species in physical and chemical traits can promote resource complementarity, allowing taxa with distinct feeding preferences and habitat requirements to coexist (Boyero et al. 2021). Additionally, water flowing from these fragments, characterized by higher levels of dissolved oxygen and lower temperatures, can enhance the overall diversity of invertebrates in impacted reaches (Goss et al. 2014; Mori et al. 2015) by providing suitable conditions for cold-water species with high oxygen demands (Boyero 2003). Together with the drift of organisms (Castro et al. 2013; Lancaster et al. 1996), forest upstream reaches might thus sustain more diverse detritus-based food webs in deforested reaches (Staudacher et al. 2018; Swan and Palmer 2006), promoting leaf breakdown and nutrient cycling. Unraveling how forest fragments help improve the condition of altered streams is a critical research gap, especially in tropical regions such as the Atlantic Forest, where deforestation occurs at alarming rates (Fundação SOS Mata Atlântica & INPE 2023).

In this study, we tested the hypothesis that forest fragments can mitigate the impacts of riparian deforestation on in-stream abiotic conditions, leaf-associated invertebrates, and leaf litter breakdown. Our objectives were (i) to evaluate the role of downstream forest fragments in buffering the negative effects of upstream riparian deforestation and (ii) to assess the potential of upstream forest fragments to alleviate impacts on deforested downstream reaches. Additionally, we investigated how subsidized leaf litter, varying in quality (recalcitrant nitrogen-fixing vs. C-labile) and diversity (monoculture vs. mixture), influences leaf-associated invertebrate assemblages and breakdown rates. We predicted forest upstream reaches (reference condition) would exhibit less impacted abiotic conditions (e.g., lower sedimentation, reduced temperature fluctuations) and more diverse invertebrate assemblages dominated by sensitive taxa than deforested upstream reaches. Because forest fragments can act as buffers, we expected to find intermediate conditions, including abiotic and biotic characteristics, in both downstream reach types, i.e., forest and deforested. These shared conditions might include greater substrate diversity and dissolved oxygen, as well as greater abundance and diversity of shredders and EPT than deforested upstream reaches. In deforested downstream reaches, we anticipated higher leaf breakdown rates due to the drift of shredders from forest upstream reaches despite the presence of taxa typical of open canopy conditions. Finally, we expected that the mixture of leaves would sustain a greater abundance and diversity of invertebrate assemblages from various functional feeding groups than would the single-leaf treatments. In addition, the invertebrate-mediated leaf breakdown rates would be higher for C-labile, high-quality leaves in both the single and mixed treatments.

## Materials and methods

### Study area

We conducted this study within the Atlantic Forest biome in Espírito Santo State, SE Brazil. The average air temperature in the study area ranges from 18 to 23 °C, with an average annual precipitation between 1,500 mm and 1,600 mm, not exceeding 60 mm in the driest month (Alvares et al. 2013). The studied stream catchments are located in mountainous areas initially covered by the Atlantic Forest and have preserved significant portions of forest fragments, surrounded by areas converted for livestock farming and single-crop plantations, such as coffee, banana, and eucalyptus.

**Fig. 1:**
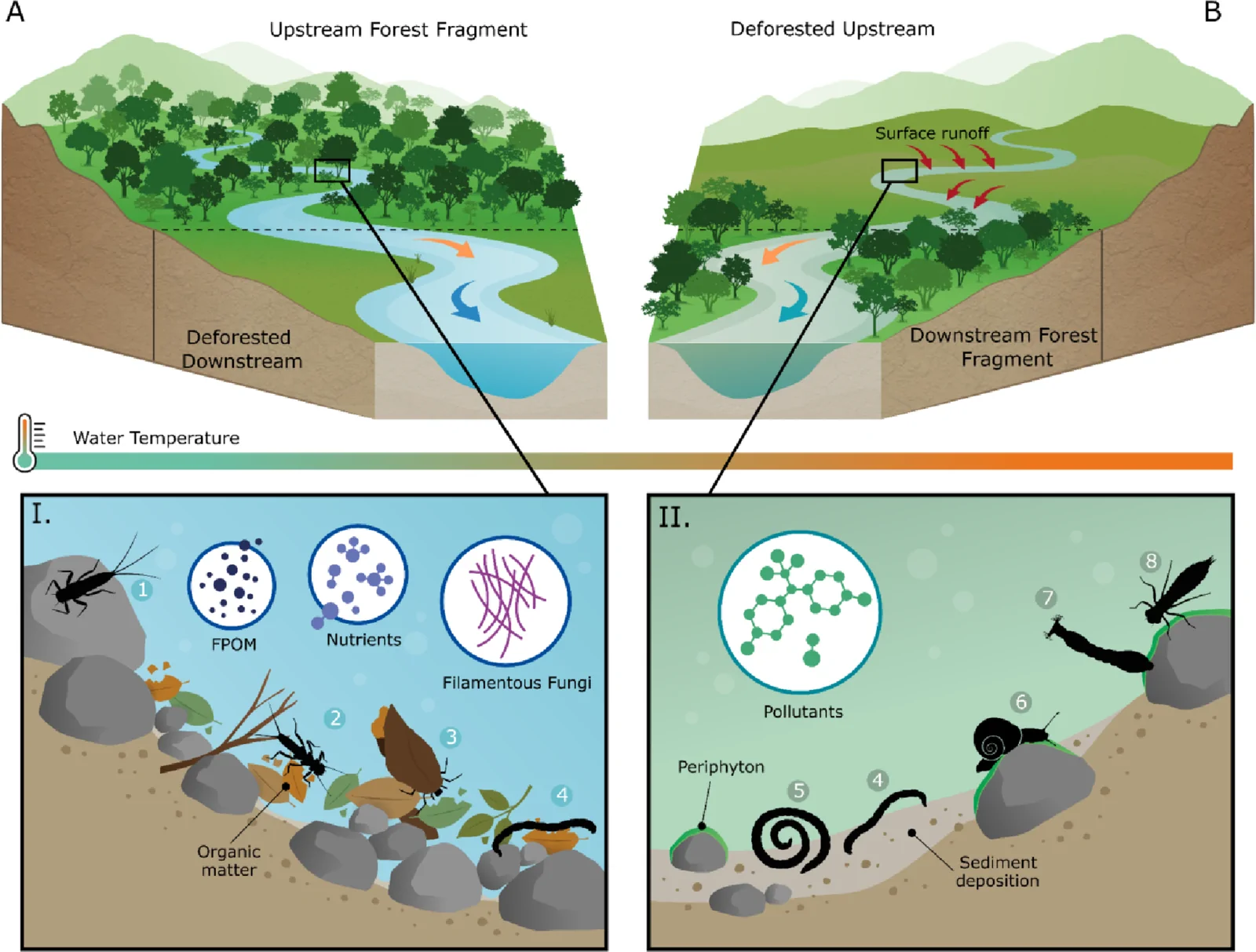
Conceptual scheme depicting headwater streams in catchments altered by land use under two different scenarios: (**A**) streams running from forest fragments (forest upstream) to deforested reaches (deforested downstream) and (**B**) streams running from deforested reaches (deforested upstream) to forest fragments (forest downstream). (I) Hypothetical representation of a stream section from a less impacted reach type (forest upstream) showing the presence of invertebrate taxa sensitive to environmental changes such as EPT (1. Ephemeroptera, 2. Plecoptera, and 3. Trichoptera) and shredders (3), as well as tolerant taxa such as Chironomids (4). Fine particulate organic matter (FPOM) and nutrients derived from organic matter decomposition mediated by microorganisms (e.g., filamentous fungi) and detritivores (e.g., shredders) were also represented. (II) Hypothetical representation of a stream section from the more impacted reach type (deforested upstream) showing the dominance of tolerant invertebrate taxa, such as collector-gatherers (5. Oligochaeta and 4. Chironomids), scrapers (6. Gastropoda), collector-filterers (7. Diptera: Simuliidae), and predators (8. Anisoptera). Note the presence of pollutants, sediment deposition from surface runoff, and periphyton growth on rocky substrates. The blue arrows denote the direction of water flux and the orange arrows denote the drift of materials and organisms

We studied 16 stream reaches located in eight stream catchments. Four of these streams flowed from the forest into the deforested reaches, whereas the other four flowed from the deforested into the forest reaches. Consequently, the studied reaches comprised four replicates of each of the four reach types: “forest upstream”, “deforested downstream”, “deforested upstream”, and “forest downstream” (Fig. 1). It is important to note that the forest reaches were inside forest fragments, as no large continuous forests were present at the study sites. We studied two reaches in each stream: one characterized by riparian vegetation with densely spaced trees and the other surrounded by deforested areas converted to pasture or agriculture. The average distance between reaches within each stream was approximately 500 m (± 250 m [SD]), with an average altitude difference of 26 m (± 26.2 m [SD]). The experiment took place from mid-May to mid-July 2021 (dry season). The upstream reaches had no other influences that would alter their condition, i.e., there was no land use above the “forest upstream” reaches and no forested fragments above the “deforested upstream” reaches.

### Reach-scale environmental characteristics

We measured water physical and chemical parameters, hydromorphology, substrate diversity, sedimentation, and riparian cover at each stream reach (Table S1). We measured the water pH, dissolved oxygen content, electrical conductivity, total dissolved solids content, and redox potential in situ using a portable multiparameter (Horiba U-50, Horiba Advanced Techno Co. Ltd., Kyoto, Japan) at the beginning, middle (30 d), and end (45–55 d) of the experiment. We also collected water samples at the beginning and end of the experiment to quantify the nitrate concentration, alkalinity, and hardness of the water. Furthermore, data loggers (HOBO Pendant Temperature/Light DataLogger; UA-002-64) were fixed on the substrate of each reach, and the water temperature was recorded every hour throughout the experiment. Based on these data, we estimated each reach’s mean daily temperature (T_mean_) and the coefficient of daily temperature variation (T_oscillation_).

We assessed the stream hydromorphology characteristics by measuring the stream width, depth, and flow velocity (using a handheld flowmeter; Flowatch, JDC Electronic SA, Yverdon-les-Bains, Switzerland) in three sections within each stream reach. To estimate the substrate diversity, we determined the proportions of different substrate categories, such as sand, gravel, cobbles, large rocks, and leaves, within a grid quadrat of 0.25 m² area (*n* = 10 quadrats per reach). We subsequently calculated the Shannon diversity index, which considers both the number of substrate types and the proportion of the total area covered by each type (*H’* = – Σ*p*_*i*_ ⋅ ln(*p*_*i*_), where *p*_*i*_ represents the proportion of the total area covered by the *i*^th^ substrate type) (Boyero 2003).

To quantify the stream sedimentation, we fixed artificial turf mats (10 × 15 cm, four replicates) on the substrate of each reach for 30 d. We then washed the turf mats individually in the laboratory over two sieves (500 and 250 μm mesh) to separate the sediment into coarse and fine particles. The samples were dried (60 °C, 48 h) and then ignited (a 250 mg subsample at 550 °C for four hours) to estimate the sedimentation of the inorganic particles per day (Von Bertrab et al. 2013). Additionally, we quantified the riparian canopy cover using fisheye images recorded at each stream reach. These images were converted to black and white, and the percentage of black pixels on the image was calculated using ImageJ (National Institutes of Health, Bethesda, Maryland, USA; version 1.52c) (Schneider et al. 2012), which serves as a proxy for riparian canopy cover.

We quantified the forest cover for each sampling point using buffers with radius of 30, 50, and 100 m centered on these points (Fig. S1). The forest cover classification was based on the native forest categories from the land use and cover mapping of Espírito Santo, conducted through the photointerpretation of Kompsat-3/3A satellite images acquired between 2019 and 2020, provided by Geobases (https://geobases.es.gov.br/). Forest cover was calculated as the proportion of forest area relative to the total buffer area, using the R programming language (R Development Core Team 2024). All geospatial data were projected in the UTM Zone 24 S coordinate system with the SIRGAS 2000 datum.

### Leaf breakdown experiment

To investigate potential variations in leaf-associated invertebrate assemblages and breakdown rates across stream reaches, we conducted a decomposition experiment using leaf litter from two native tree species. We used naturally fallen leaves of *Miconia chartacea* Triana (Melastomataceae), a C-labile, fast-decomposing leaf species, and *Inga laurina* (Sw.) Willd. (Fabaceae), an N-fixing, slow-decomposing leaf species. We also chose these species to test the effects of resource quality because combining different functional types might result in diverse effects on breakdown rates (Handa et al. 2014). Both tree species are commonly found near the study reaches and are widely distributed in the Atlantic Forest (Casotti et al. 2019; Kiffer et al. 2018). To characterize the leaf species thoroughly, we analyzed the initial chemical composition of both leaves (time 0). We determined the leaf nitrogen, phosphorus, and potassium (NPK) concentrations; total phenolic content; toughness; and fiber, lignin, and cellulose concentrations (via the gravimetric method) (Bärlocher et al. 2020). We used four analytical replicates per leaf species for each analysis (Table S2, Fig. S2).

To estimate the leaf breakdown rates and invertebrate colonization, we incubated leaves of *M. chartacea* and *I. laurina* in single and mixed treatments within coarse (10 mm) and fine (0.25 mm) mesh litter bags to allow or exclude invertebrates from accessing the leaves, respectively. Different mesh sizes allowed us to better understand the relative roles of microbial and invertebrate decomposers in leaf litter decomposition under various experimental conditions (Frainer et al. 2021). For the single-species treatments, we placed 5.00 ± 0.05 g (air dry weight) of one of the studied leaves into each litter bag. In the mixed treatment, we combined 2.50 ± 0.05 g from each leaf species in the same litter bag. We used four replicates per treatment in each reach, totaling 384 litter bags (3 leaf treatments × 2 mesh sizes × 4 replicates × 16 stream reaches).

We removed all of the litter bags from the streams when approximately 50% of the initial mass of the fast-decomposing species (i.e., *M. chartacea*) had decomposed, which occurred after 45–55 d. We used additional control litter bags to determine the end of the experiment in each stream reach. We removed the litter bags from the streams and placed them individually in plastic bags. In the laboratory, we washed the leaves with a 250 μm mesh sieve to retain the associated invertebrates, which were preserved in ethanol (70%). They were later counted and identified with a stereo microscope (ZEISS Stemi 305, 32× magnification) to the lowest taxonomic level using identification keys (Hamada et al. 2014, 2018; Mugnai et al. 2010). The functional feeding groups of aquatic insects were based on Baptista et al. (2006); Oliveira and Nessimian (2010); Ramírez and Gutiérrez-Fonseca (2014). We found no invertebrates in fine-mesh litter bags. Finally, we oven-dried (60 °C, 72 h) the remaining leaves from the coarse- and fine-mesh litter bags to calculate the leaf mass loss and breakdown rates. We excluded data from litter bags with more than 80% leaf mass loss (11 litter bags in total), as invertebrate colonization and activity might be reduced.

### Statistical analyses

Our statistical approach comprised three primary steps. First, we characterized stream reaches based on multiple environmental variables that are fundamental to the biodiversity and functioning of stream ecosystems (see Table S1). We excluded the parameters that did not differ among stream reaches from the analysis, as such parameters did not contribute to environmental characterization. Because some environmental variables were collected multiple times, we first calculated the mean values of each descriptor at the reach level. We then normalized these environmental variables by subtracting their means and dividing them by their standard deviations. Before performing the analyses, we conducted Pearson correlations to exclude ecologically redundant variables (*r* > 0.75). To investigate potential variations in stream environmental characteristics based on reach type (i.e., forest upstream, forest downstream, deforested downstream, and deforested upstream), we conducted a permutational multivariate analysis of variance (PERMANOVA) (Anderson 2001) via the *adonis* function from the *vegan* package (Oksanen et al. 2024) in R (version 4.3.1; R Development Core Team 2024). We calculated the dissimilarity as Euclidean distances with 999 permutations and included the stream as a “strata” to account for the lack of independence between reaches within the same stream. Additionally, we conducted a principal component analysis (PCA) to visualize the ordination of the sampling reaches according to the stream characteristics. We selected the axes using the broken stick model, where a given axis is retained if its observed eigenvalues exceed the expected eigenvalues generated by the broken stick (Jackson 1993).

In the second step, we assessed the influence of stream reach type and leaf species (mixed treatment, *M. chartacea*, and *I. laurina*) on the leaf-associated invertebrate assemblages. Initially, we analyzed the differences in assemblage composition via PERMANOVA, employing the *adonis* function from the *vegan* package (Oksanen et al. 2024), with dissimilarity calculated as the Bray‒Curtis distance in Hellinger-transformed community data (leaf litter bags of each treatment per reach pooled). We assessed group dispersion homogeneity (reach type and leaf species) via PERMDISP with the *betadisper* function (Oksanen et al. 2024). We analyzed the community data in relation to the reach types and leaf treatments, including streams as strata. Significant results were tested by pairwise comparisons using the function *pairwise.adonis2* (“*pairwiseAdonis*” package) (Arbizu 2020). We used non-metric multidimensional scaling (NMDS) to visualize differences in the invertebrate assemblage composition. We then performed an indicator value analysis (IndVal) (Dufrêne and Legendre 1997) to identify taxa that are associated to each reach type using the function *indval* in the *labdsv* package (Roberts 2023). IndVal combines the mean abundance of a species with its frequency of occurrence within each group. A high indicator value exists when a species is present in most reaches of a given type (fidelity). The indicator value ranges from 0 to 1, with 1 indicating a perfect indicator taxon.

Using generalized linear mixed models (GLMMs), we then examined the impact of stream reach type and leaf species treatments on the abundance and diversity of EPT taxa (abundance: negative binomial, link = log; diversity: Gaussian, link=identity) and assemblages of shredders (abundance and diversity: Tweedie, link = log), collector-gatherers (abundance: negative binomial, link=logit; diversity: Gaussian, link=identity), collector-filterers (abundance and diversity: Tweedie, link = log), scrapers (abundance and diversity: Tweedie, link = log), and predators (abundance: negative binomial, link = log; diversity: Gaussian, link=identity). We used the negative binomial distribution for overdispersed count data and the Tweedie distribution for non-negative continuous data characterized by a point mass at zero and a right-skewed positive distribution. Model significance was evaluated using Type II Wald χ² tests. To facilitate biological interpretation of the model results, we estimated marginal means (EMMs) and their 95% confidence intervals for each level of stream reach type and leaf species using the *emmeans* package (Lenth 2024). All estimates were reported on the response scale. For decomposition models fitted using square-root-transformed decomposition rates to improve model assumptions, estimated marginal means and confidence intervals were back-transformed to the original scale prior to reporting. We addressed pseudo replication by treating streams as a random factor (Tables S4 and S6).

In the third step, we investigated how the leaf breakdown rates in the “*Miconia* single”, “*Miconia* mixed”, “*Inga* single”, and “*Inga* mixed” treatments varied according to the interaction between stream reach type and the presence of invertebrates (comparing coarse vs. fine mesh sizes) using GLMMs (Table S5). We estimated the leaf breakdown rates using the negative exponential model and the remaining mass over time (Webster and Benfield 1986):

$$ M_{t} = M_{0} e^{{ - kt}} $$

where *M*_*t*_ = remaining mass, *M*_0_ = initial mass, *k* = breakdown rate, and *t* = incubation time. We expressed *k* in degree days (dd^− 1^) by computing the thermal sums for each stream based on the average daily temperatures calculated from the hourly records (Gessner and Peeters 2020). In each GLMM model, we modeled the breakdown rate as a function of the interaction between reach type (i.e., forest upstream, forest downstream deforested upstream, deforested downstream) and mesh size (i.e., coarse vs. fine), including streams as a random factor.

We provided detailed information on the model structure, family distribution, and link functions for all GLMMs in Tables S4, S6, and S7. To assess the conformity of the model predictions with the data, we used the *simulateResiduals* function from the *DHARMa* package (Hartig 2022), fitting models to simulate 1,000 datasets for each. We subsequently conducted a Kolmogorov–Smirnov test on the simulated residuals and observed the residuals to assess the uniformity. As part of the model validation process, we employed the *testDispersion* function from the *DHARMa* package to conduct a simulation-based test for over- and underdispersion. All analyses were performed using R (R Development Core Team 2024).

## Results

### Reach-scale environmental characteristics

**Fig. 2:**
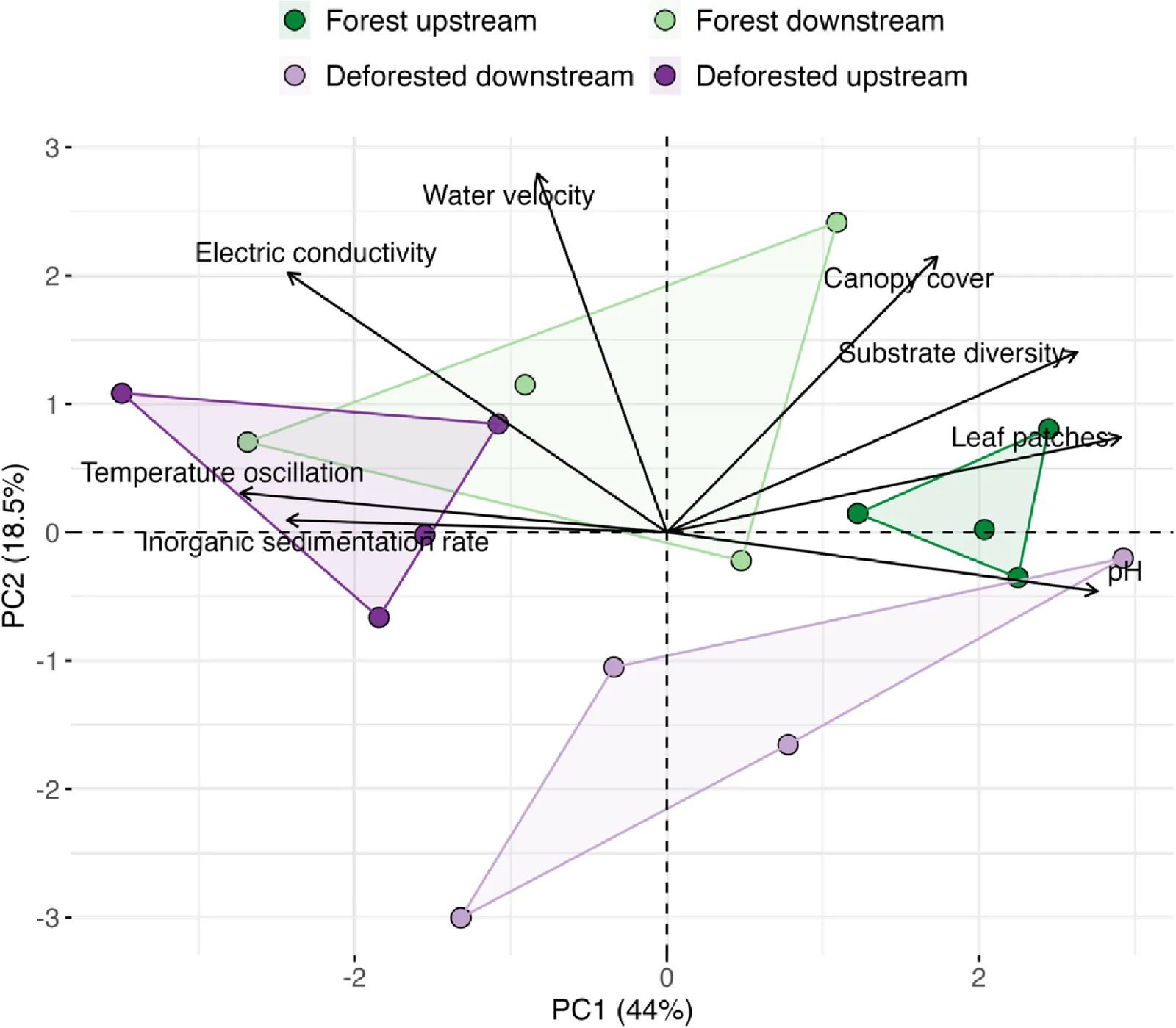
Principal component analysis (PCA) of the environmental characteristics measured at 16 stream reaches in catchments altered by land use. The different reach types include forests upstream (dark green), forests downstream (light green), deforested downstream (light purple), and deforested upstream (dark purple). We only show environmental characteristics with correlations of *r* > 0.5 and *p* < 0.05. The PCA was calculated with data from 9 variables from 16 stream reaches

The composition of the environmental characteristics varied according to the stream reach type (PERMANOVA pseudo-*F* = 3.28, *R*^2^ = 0.45, *p* = 0.01). The first two axes of the PCA explained 64.3% of the total variance in the environmental characteristics of the stream reaches (Fig. 2). The first principal component (PC1) of the PCA accounted for 39.6% of the variance in stream characteristics and was positively associated with pH, percentage of substrate covered by leaf patches, substrate diversity, and canopy cover (Fig. 2, Table S3). Conversely, PC1 was negatively associated with daily temperature oscillation, the inorganic sedimentation rate, and electrical conductivity. PC1 distinguished the forest upstream (right side of PC1) from the deforested upstream and forest downstream reaches (left side of PC1). Forest cover, electrical conductivity, canopy cover, and water velocity were positively correlated with the second component, explaining 24.7% of the environmental variation (Fig. 2, Table S3). PC2 distinguished forest downstream (upper side of PC2) from deforested downstream reaches (bottom side of the PC2). As predicted, the results from PERMANOVA and PCA indicated that the environmental characteristics of forest upstream reaches differed from those of deforested upstream reaches. In contrast, forest downstream and deforested downstream reaches also differed, but showed intermediate conditions relative to the more (deforested upstream) and less (forest upstream) impacted reaches.

### Leaf-associated invertebrates

**Fig. 3:**
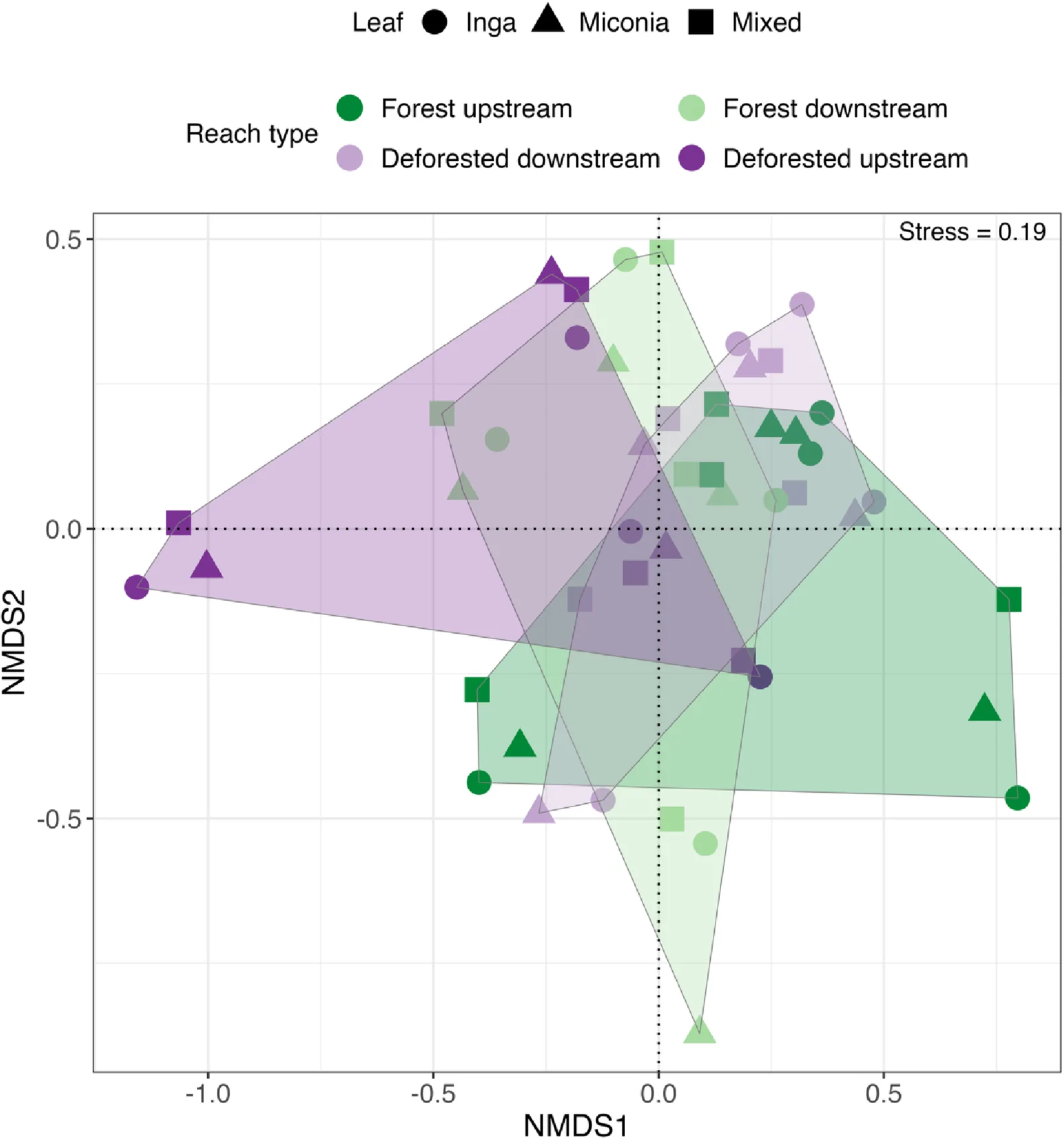
Non-metric multidimensional scaling (NMDS) ordination of invertebrate assemblages associated with leaves of *Inga laurina* (circles), *Miconia chartacea* (triangles), and their mixtures (squares). Ordination was based on a Bray–Curtis dissimilarity matrix calculated from the abundance of 131 invertebrate taxa across 47 leaf-pack assemblages (*N* = 47) collected from 16 reaches in eight streams. The assemblages are situated at stream reaches characterized by different reach types: forest upstream (dark green), forest downstream (light green), deforested downstream (light purple), and deforested upstream (dark purple). The stress value is shown within the figure

The taxonomic composition of the invertebrate assemblages differed among stream reaches but not among the leaf treatments (PERMANOVA; leaf treatments: *R*^2^ = 0.01, *F* = 0.38, *p* = 0.89; stream reaches: *R*^2^ = 0.18, *F* = 3.18, *p* < 0.001; Fig. 3). The taxonomic compositions in assemblages in the forest upstream and deforested downstream reaches (pairwise comparison: *F* = 1.70, *R*^2^ = 0.07, *p*-adjusted = 0.55) and in the deforested upstream and forest downstream reaches were similar (pairwise comparison: *F* = 2.01, *R*^2^ = 0.08, *p*-adjusted = 0.29). However, significant differences were found between the forest upstream and deforested upstream reaches (F = 4.75, *R*^2^ = 0.18, *p*-adjusted = 0.003) and between forest upstream and forest downstream reaches (*F* = 3.23, *R*^2^ = 0.12, *p*-adjusted = 0.008). A total of 19 significant indicator taxa was detected: three for the forest upstream reaches, two for the forest downstream reaches, five for the deforested downstream reaches, and nine for the deforested upstream reaches (Table 1). Overall, 28,465 invertebrate individuals representing 135 taxa were collected across all leaf treatments and stream reaches (Fig. S3).

**Table 1:** Results of the Indval analysis of aquatic invertebrate assemblages associated with leaves of *Miconia chartacea* and *Inga laurina* in single- and mixed-leaf treatments at different stream reach types (i.e., forest upstream, forest downstream, deforested downstream, and deforested upstream)

Reach type	Taxon	Indval	*p*
Forest upstream	Leptophlebiidae (Cg)	0.34	0.01
	*Triplectides* (Sh)	0.33	0.03
	*Tricorythopsis* (Sc)	0.31	0.04
Forest downstream	*Phylloicus* (Sh)	0.41	0.03
	Gomphidae (P)	0.28	0.03
Deforested downstream	Libellulidae (P)	0.48	0.01
	*Tricorythodes* (Sc)	0.47	0.01
	*Oxyethira* (Sc)	0.47	0.01
	Baetidae (Sc)	0.43	0.04
	*Massartella* (Cg)	0.25	0.04
Deforested upstream	Simuliidae (Cf)	0.78	0.003
	Ostracoda (Cf)	0.78	0.01
	Planaria (P)	0.76	0.001
	Smicridea (Cf)	0.64	0.001
	Hydroptilidae (Sc)	0.62	0.002
	*Hydroptila* (Sc)	0.45	0.02
	Nematoda (P)	0.28	0.02
	Hirudinea (P)	0.27	0.01
	*Melanoides* (Sc)	0.24	0.03

The functional feeding groups are indicated by the following codes: collector-filterers (Cf), collector-gatherers (Cg), scrapers (Sc), shredders (Sh), and predators (P). The indicator value ranges from 0 to 1, with 1 indicating a perfect indicator taxon

**Fig. 4:**
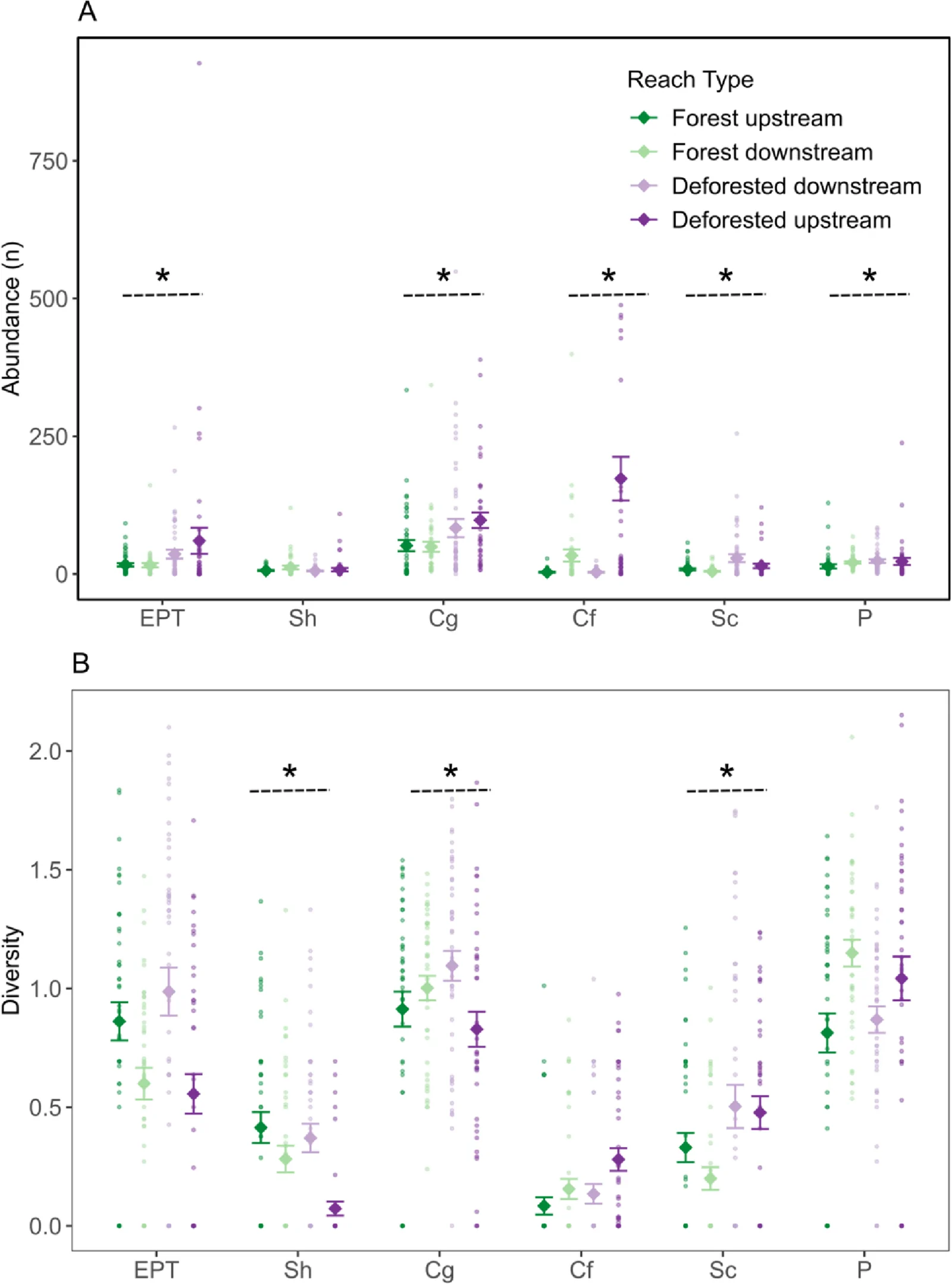
Values (mean ± SE) of the abundance (**A**) and Shannon diversity (**B**) of invertebrate functional feeding groups and EPT taxa in the different reach types (forests upstream [dark green], forests downstream [light green], and deforested upstream [light purple]; N = 172 litter bags). The dotted bars with an asterisk (*) above the groups denote significant differences (*p* < 0.05) assessed separately for each invertebrate group. ‘EPT’ = Ephemeroptera, Plecoptera, and Trichoptera; ‘Sh’ = shredders; ‘Cg’ = collector-gatherers; ‘Cf’ = collector-filterers; ‘Sc’ = scrapers; ‘P’ = predators

There was an increase in the abundance of most functional feeding groups from the least impacted (forest upstream) to the most impacted stream reach type (deforested upstream), except for shredders (Table 2; Fig. 4A). This trend particularly reflected the substantial increase in the abundance of collector-filterers in the deforested upstream reaches compared with the forest upstream reaches, with an average fortyfold increase (Fig. 4A, Table S5). Notably, the abundance of collector-filterers in the deforested downstream reaches resembled that in the forest upstream reaches (Table 2 and S4, Fig. 4A). The abundance of collector-filterers was ten times greater in the forest downstream reaches than in the forest upstream reaches (Fig. 4A, Table S5). Additionally, there was a trend toward greater collector-gatherer abundance in the forest upstream reaches than in the deforested upstream reaches (Fig. 4A, Table S4). Moreover, there was a lower collector-gatherer abundance in the forest downstream reaches than in the deforested upstream reaches (estimates − 0.74, *Z* = -3.58, *p* = 0.001). The number of scrapers was approximately three times greater in the deforested downstream reaches than in the forest upstream reaches (Table 2 and S4, Fig. 4A). The data revealed a lower abundance of predators in the forest upstream reaches than in the deforested downstream reaches (Fig. 4A, Table S4). Finally, statistical analyses did not detect differences in EPT abundance between the forest upstream reach and the other three reach types (Table S4). However, EPT abundance was six times higher in the deforested upstream reaches than in the forest downstream reaches (estimate = -0.98, *Z* = -2.29, *p* = 0.02; Table 2; Fig. 4A).

**Table 2:** Summary of the generalized linear mixed models (GLMMs) evaluating the effects of stream reach type (forest upstream, forest downstream, deforested downstream, and deforested upstream) and leaf treatment (*Miconia chartacea*, *Inga laurina*, and mixed leaves) on the abundance and diversity of EPT taxa and functional feeding groups

Response	Predictor	χ²	*p*	Treatment	Estimated marginal mean (95% CI)
Abundance					
Shredders	Reach type	5.07	0.17	—	—
	Leaf	21.1	**< 0.001**	Miconia	7.08 (3.31–15.11)
				Mixed	4.97 (2.32–10.68)
				Inga	3.21 (1.49–6.91)
Collector-gatherers	Reach type	17.63	**< 0.001**	Forest upstream	31.70 (13.70–73.60)
				Forest downstream	46.40 (19.90–108.50)
				Deforested downstream	45.80 (19.90–105.70)
				Deforested upstream	98.00 (42.30–226.90)
	Leaf	0.31	0.86	—	—
Collector-filterers	Reach type	68.41	**< 0.001**	Forest upstream	2.13 (0.62–7.35)
				Forest downstream	17.23 (5.34–55.61)
				Deforested downstream	1.99 (0.58–6.84)
				Deforested upstream	83.26 (26.26–263.99)
	Leaf	1.86	0.39	—	—
Scrapers	Reach type	30.84	**< 0.001**	Forest upstream	3.43 (0.79–14.90)
				Forest downstream	4.16 (0.97–17.90)
				Deforested downstream	8.62 (2.01–36.90)
				Deforested upstream	10.77 (2.54–45.60)
	Leaf	0.09	0.954	—	—
Predators	Reach type	16.66	**< 0.001**	Forest upstream	8.84 (4.27–18.30)
				Forest downstream	22.72 (10.96–47.10)
				Deforested downstream	17.16 (8.34–35.30)
				Deforested upstream	19.98 (9.67–41.30)
	Leaf	0.69	0.71	—	—
EPT	Reach type	14.06	**0.003**	Forest upstream	12.10 (3.95–37.40)
				Forest downstream	14.10 (4.52–43.70)
				Deforested downstream	14.50 (4.75–44.60)
				Deforested upstream	39.60 (12.82–122.30)
	Leaf	0.17	0.92	—	—
Diversity					
Shredders	Reach type	9.27	**0.026**	Forest upstream	0.367 (0.166–0.811)
				Forest downstream	0.222 (0.094–0.527)
				Deforested downstream	0.301 (0.135–0.671)
				Deforested upstream	0.063 (0.021–0.187)
	Leaf	4.04	0.132	—	—
Collector-gatherers	Reach type	8.01	0.05	Forest upstream	0.938 (0.593–1.280)
				Forest downstream	1.004 (0.659–1.350)
				Deforested downstream	1.094 (0.750–1.440)
				Deforested upstream	0.848 (0.503–1.190)
	Leaf	0.95	0.621	—	—
Collector-filterers	Reach type	6.32	0.097	—	—
	Leaf	1.62	0.444	—	—
Scrapers	Reach type	15.05	**0.002**	Forest upstream	0.131 (0.017–1.030)
				Forest downstream	0.089 (0.011–0.704)
				Deforested downstream	0.179 (0.023–1.394)
				Deforested upstream	0.211 (0.027–1.642)
	Leaf	1.73	0.422	—	—
Predators	Reach type	3.89	0.273	—	—
	Leaf	3.02	0.221	—	—
EPT	Reach type	2.44	0.487	—	—
	Leaf	0.96	0.62	—	—

Model significance was assessed using Type II Wald χ² tests. Estimated marginal means (EMMs) and their 95% confidence intervals are presented only for significant effects and are reported on the response scale. Empty cells indicate predictors with no statistically significant effect (*p* > 0.05), for which estimated marginal means are not presented

There was no evidence of an effect of stream reach type on shredder abundance (Table 2; Fig. 4A). However, the leaf treatment significantly affected their abundance (Table 2 and S4, Fig. S4). Specifically, shredders were more abundant in the leaf litter bags containing *M. chartacea* alone than in those containing *I. laurina* alone or in the mixed treatment (Table S4; Fig. S4). The abundance of all other functional feeding groups and EPT was not influenced by leaf treatment (Table S4). However, there was evidence that the stream reach type affected the diversity of functional feeding groups, specifically shredders, collector-gatherers, and scrapers (Table 2 and S6, Fig. 4B). The shredders presented lower diversity in the deforested upstream reaches (Table S6, Fig. 4B), whereas the scrapers presented higher diversity in the deforested upstream reaches than in the forest downstream reaches (estimate = -0.89, *Z* = -3.70, *p* = 0.001). Compared with the forest upstream reaches, the collector-gatherers presented greater diversity in the deforested downstream reaches (estimate = 0.16, *t* = 2.01, *p* = 0.046; Fig. 4B).

### Leaf breakdown rates

**Fig. 5:**
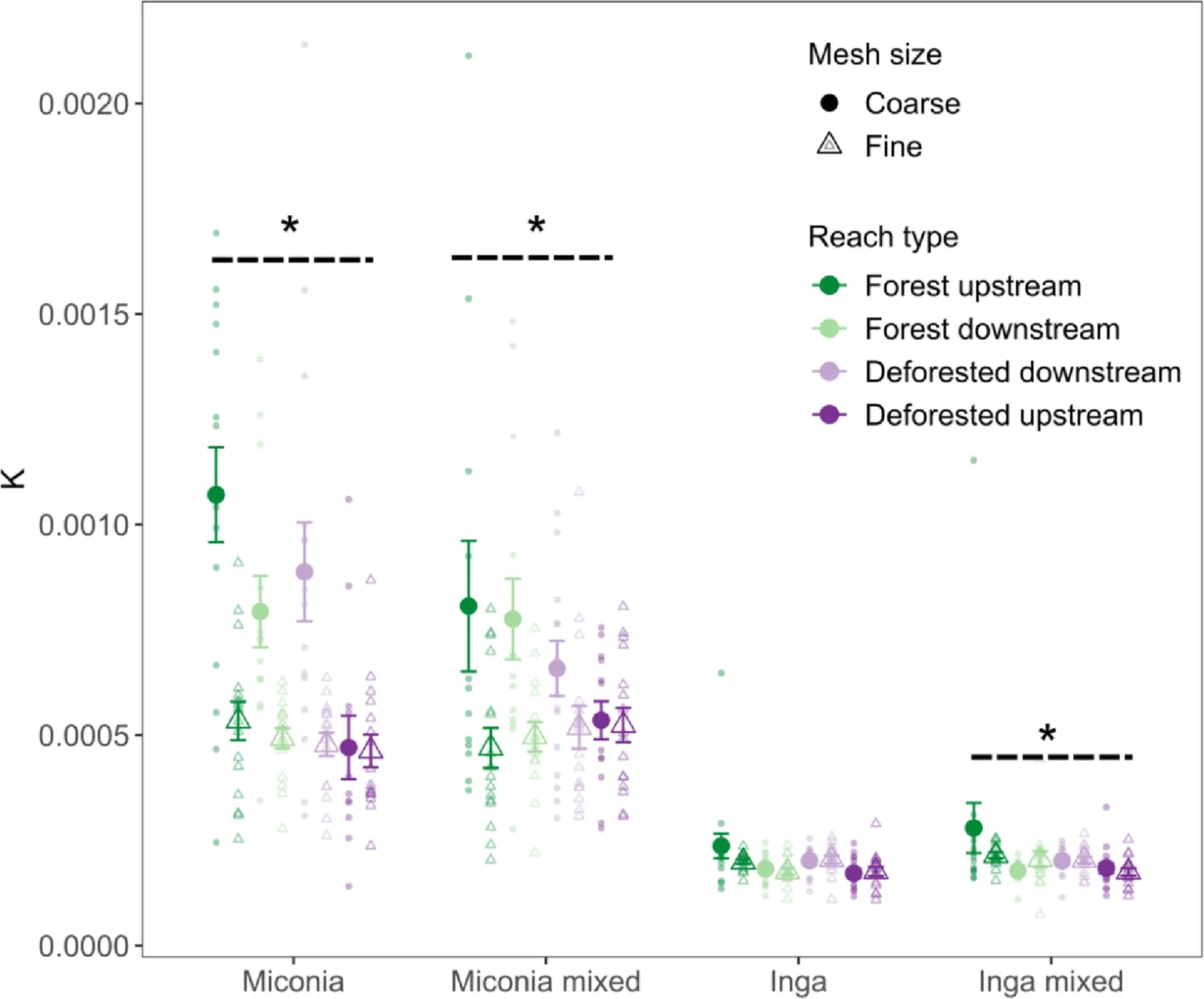
Leaf breakdown rates (*k* [mg dd^− 1^]; mean ± SE) of leaves of *Miconia chartacea* and *Inga laurina* incubated in single and mixed treatments depending on the presence of invertebrates (coarse vs. fine mesh size) and reach type (forest upstream [dark green], forest downstream [light green], deforested upstream [light purple], and deforested downstream [dark purple]; *N* = 492 litter bags: 238 coarse and 254 fine mesh bags). The continuous bars (indicating the effect of reach type) and dotted bars (indicating the interactive effect of invertebrate presence (mesh size×reach type) with an asterisk (*) above the groups denote significant differences (*p* < 0.05) assessed separately for each leaf treatment

Leaf breakdown rates varied as a function of leaf treatment, the presence of invertebrates (i.e., coarse- vs. fine-mesh bags), and stream reach type (Fig. 5, Table S7). On average, *M. chartacea* leaves presented breakdown rates three times greater than those of *I. laurina* leaves (*k*_*Miconia* single_ = 0.000648 ± 0.000354 dd^− 1^; *k*_*Miconia* mixed_ = 0.000591 ± 0.000288 dd^− 1^; *k*_*Inga* single_ = 0.000193 ± 0.000055 dd^− 1^; *k*_*Inga* mixed_ = 0.000205 ± 0.000095 dd^− 1^). There was substantial evidence that invertebrates contributed to the processing of *M. chartacea* leaves in both the single and mixed treatments (Table 3 and S7, Fig. 5); however, this contribution was contingent on the reach type (Table 3 and S7, Fig. 5). In the coarse mesh bags, the leaf breakdown rates of *M. chartacea* were higher in the forest upstream reaches than in deforested upstream reaches (Fig. 5). With respect to *I. laurina*, there was no evidence of an interaction between reach type and the presence of invertebrates (mesh size) on leaf breakdown in either the single or mixed treatments (Table 3 and S7, Fig. 5). However, the reach type affected leaf breakdown in the mixed treatments, with higher rates observed in forest upstream reaches (Table 3 and S7, Fig. 5). In fine mesh bags, the leaf breakdown rates did not vary among stream reach types for either *M. chartacea* or *I. laurina* (Fig. 5).

**Table 3:** Summary of the generalized linear mixed models (GLMMs) evaluating the effects of reach type (forest upstream, forest downstream, deforested downstream, and deforested upstream), mesh size (coarse vs. fine), and their interaction on leaf breakdown rates (*k*) of *Miconia chartacea* and *Inga laurina* in the single- and mixed-leaf treatments

Leaf treatment	Predictor	χ²	*p*	Treatment Combination	Estimated marginal mean k (95% CI)
Miconia single	Reach type	25.3	< 0.001	—	—
	Mesh size	44.24	< 0.001	—	—
	Reach type × Mesh size	19.47	< 0.001	Forest upstream – Coarse	0.001038 (0.000761–0.001358)
				Forest downstream – Coarse	0.000789 (0.000548–0.001074)
				Deforested downstream – Coarse	0.000836 (0.000590–0.001124)
				Deforested upstream – Coarse	0.000471 (0.000288–0.000699)
				Forest upstream – Fine	0.000519 (0.000330–0.000751)
				Forest downstream – Fine	0.000488 (0.000305–0.000713)
				Deforested downstream – Fine	0.000471 (0.000292–0.000694)
				Deforested upstream – Fine	0.000451 (0.000276–0.000669)
Miconia mixed	Reach type	8.58	0.035	—	—
	Mesh size	16.9	< 0.001	—	—
	Reach type × Mesh size	7.9	0.048	Forest upstream – Coarse	0.000787 (0.000578–0.001028)
				Forest downstream – Coarse	0.000750 (0.000551–0.000979)
				Deforested downstream – Coarse	0.000634 (0.000455–0.000843)
				Deforested upstream – Coarse	0.000552 (0.000382–0.000755)
				Forest upstream – Fine	0.000452 (0.000303–0.000630)
				Forest downstream – Fine	0.000488 (0.000332–0.000675)
				Deforested downstream – Fine	0.000503 (0.000345–0.000690)
				Deforested upstream – Fine	0.000512 (0.000353–0.000701)
Inga single	Reach type	4.58	0.206	—	—
	Mesh size	0.77	0.381	—	—
	Reach type × Mesh size	3.38	0.337	—	—
Inga mixed	Reach type	9.7	0.021	Forest upstream	0.000234 (0.000189–0.000285)
				Forest downstream	0.000188 (0.000148–0.000234)
				Deforested downstream	0.000201 (0.000159–0.000248)
				Deforested upstream	0.000178 (0.000138–0.000222)
	Mesh size	3.71	0.054	—	—
	Reach type × Mesh size	4.85	0.183	—	—

Model significance was assessed using Type II Wald χ² tests. Estimated marginal means (EMMs) and their 95% confidence intervals are presented only for significant effects. For models with significant reach type × mesh size interactions, EMMs are shown for each combination of factors; when only a main effect was significant, EMMs are averaged across the levels of the other factor. Empty cells indicate predictors with no statistically significant effect (*p* > 0.05), for which estimated marginal means are not presented. Each model includes three degrees of freedom for the reach type, one degree of freedom for the mesh size, and three degrees of freedom for the reach × mesh size

## Discussion

This study demonstrates that the presence of forest fragments in agricultural catchments benefits water quality, invertebrate communities, and leaf litter breakdown, thereby contributing to the resilience of stream ecosystems to land-use changes. In addition, our results support the idea that the buffering capacity of forest riparian zones could extend to deforested downstream reaches. This finding could be a consequence of a reduced sediment load, a moderate stream temperature, and increased habitat complexity. Similarly, our findings emphasize the importance of downstream forest fragments in mitigating the adverse effects of upstream riparian deforestation and the relatively fast recovery of communities after an impacted stream enters a forested section. The forest downstream reaches shared similarities with the forest upstream reaches, including a greater diversity of shredders and a lower abundance of scrapers than the deforested upstream reaches did. Some of these differences were also reflected in the functioning of the stream ecosystems. Compared with deforested reaches, forest reaches presented higher breakdown rates of fast-decomposing leaf species, underscoring the significant influence of forest fragments on crucial ecosystem processes.

### Reach-scale environmental characteristics

In this study, we observed pronounced environmental differences between upstream forest and deforested reaches, highlighting the potential negative impact of riparian forest deforestation on stream ecosystems (Baptista et al. 2006; Marques et al. 2021; Machado-Silva et al. 2022; Oester et al. 2023). For example, the absence of forest cover in upstream reaches is correlated with substrate homogenization, including the lack of leaf patches on the streambed, a critical food and habitat source, especially for detritivores (Anderson and Cummins 1979; Kobayashi and Kagaya 2004). Moreover, there was a noticeable increase in water temperature oscillations and sediment deposition, all of which impose severe threats to taxa sensitive to environmental change (Berger et al. 2017; Bonacina et al. 2023). Land-use change has been widely reported to reduce habitat quality in streams, contributing to biodiversity decline (Brauns et al. 2022; Dala-Corte et al. 2020). However, our study suggests that both upstream and downstream forest fragments can reduce the impacts of riparian forest removal on stream characteristics. According to the predicted impact gradient, the downstream forest and deforested reaches presented environmental characteristics that were intermediate to those found in the most preserved and impacted reaches. Some evidence suggests that forest fragments can reset the water quality in deforested streams (Fernandes et al. 2014; Harding et al. 2006). Although we did not observe a complete recovery of stream reaches from deforestation, our results suggest the potential for such improvements to enhance stream conditions over relatively short distances along the river system, leading to positive consequences for aquatic biodiversity.

### Leaf-associated invertebrates

Riparian deforestation is generally associated with increased runoff, alterations to physical habitats, and changes in water quality, which can lead to highly altered invertebrate assemblages, reflecting a heterogeneous loss of diversity and the persistence of tolerant or resistant species (Silva-Araújo et al. 2020). However, we found similar taxonomic compositions of leaf-associated invertebrate assemblages between our reference reach type, i.e., the forest upstream reach and the deforested downstream reach. This similarity is likely a consequence of the downstream dispersal of aquatic invertebrates through drift (Elliott 2003; Lancaster et al. 1996), highlighting the importance of preserving forest upstream fragments in agricultural catchments. Despite these similarities in taxonomic composition, the taxa that are indicators of deforested downstream reaches are usually associated with streams under intermediate impact levels, such as *Oxyethira* sp. and Baetidae (Nessimian et al. 2008), or preserved reaches, such as *Massartella* sp. (Huiñocana et al. 2020). While the presence of upstream forests improves stream ecosystem conditions, the occurrence of more tolerant species in deforested downstream reaches suggests the persistence of some level of anthropogenic impact.

The drift of tolerant invertebrate taxa might also explain the small differences in invertebrate composition between the deforested upstream reaches and the forest downstream reaches. Nevertheless, the forest downstream reaches had more suitable habitat conditions than the deforested reaches did, as evidenced by the presence of larvae of *Phylloicus* sp. Both *Phylloicus* sp. and *Triplectides* sp., which were indicators of forest reaches, are caddisfly genera typically associated with leaf patches in preserved stream ecosystems (Huiñocana et al. 2020; Siqueira et al. 2015). Conversely, almost all indicator taxa of deforested upstream reaches are organisms commonly found in highly impacted lotic ecosystems, such as dipterans (Simuliidae; Kasangaki et al. 2008), ostracods (Schmitz et al. 2024), tolerant caddisflies (*Smicridea* sp. and *Hydroptila* sp.; Huiñocana et al. 2020), and invasive gastropods of the genus *Melanoides*. Therefore, our findings corroborate previous research indicating that riparian deforestation negatively impacts invertebrate assemblages by reducing the relative abundance of sensitive taxa at the expense of tolerant taxa (Dala-Corte et al. 2020; Oester et al. 2023).

In addition to changes in assemblage composition, our study revealed an increase in the relative abundance of leaf-associated invertebrates across most functional feeding groups, from the reference to the most impacted stream reach type. Functional feeding groups are defined by traits that indicate mechanisms of food acquisition and reveal how well a species can adapt to varying environmental conditions, including changes in food resources (Boyero 2005; Voigt et al. 2007). The vulnerability of organisms within a feeding group is determined by how feeding traits correlate with resistance traits (Suding et al. 2008). While our study did not consider resistance traits, it is worth noting that collector-filterers, collector-gatherers, and scrapers are reportedly less sensitive to disturbances, thriving in open canopy environments with high levels of anthropogenic impact (Addo-Bediako 2021; Barbour et al. 1996; Jiang et al. 2011). Moreover, removing riparian forests increases surface runoff, resulting in sediment deposition and FPOM transport from terrestrial ecosystems (Tabacchi et al. 2000). Additionally, these reaches often have greater periphyton growth (also on leaves in our case) because of less shading. These environmental characteristics provide resources for collector-gatherers, collector-filterers, and scrapers and could explain the relatively high abundances of these functional feeding groups in deforested upstream reaches (Burrows et al. 2021; Lima et al. 2022). In turn, the higher abundance of collector-filterers in the forest downstream reaches could be attributed to the downstream drift of organisms and food items. On the other hand, the greater abundance and diversity of predators in deforested reaches may be associated with the higher abundance of potential prey, particularly small, soft-bodied collector-gatherer and scraper taxa (Elton and Elton 1927; Friman et al. 2008). Because we did not quantify biomass, this pattern should not be interpreted as evidence of greater energy availability.

Despite being considered indicators of high environmental quality (Oliveira et al. 2019; Silva-Araújo et al. 2020; Tubić et al. 2024), EPTs and shredders did not show higher abundances in the forest upstream reaches. However, we observed a predominance of tolerant EPT taxa, such as Baetidae, *Hydroptilla* sp., and *Oxyetira* sp., in stream reaches lacking riparian vegetation. These findings suggest that reach type affects the abundances of sensitive and tolerant taxa but not the total invertebrate abundance in the studied streams. On the other hand, the reduced diversity of shredders in deforested reaches is a clear indicator of environmental degradation (Hieber and Gessner 2002; Masese et al. 2014). In forest headwater streams, shredders primarily feed on allochthonous leaves (Neres-Lima et al. 2017), which makes them particularly sensitive to changes in riparian zones, including deforestation (Masese et al. 2014; Silva-Araújo et al. 2020). Therefore, lightly and moderately impacted reaches likely support greater shredder diversity because of the high availability of diverse and abundant food resources and favorable environmental conditions (Kobayashi and Kagaya 2004). These results highlight the critical role of maintaining riparian vegetation and diverse habitat structures (i.e., habitat heterogeneity) to support shredders and their essential ecological functions in stream ecosystems.

Contrary to our expectations, we did not observe an increased abundance or diversity of invertebrates in the mixed-leaf treatment. Shredders, however, were more abundant and had lower diversity in the single treatments of *M. chartacea* and *I. laurina*. These findings suggest that leaf quality is more important than leaf diversity for the colonization of leaves by shredders (Graça and Zimmer 2020; Sena et al. 2020). In addition, shredder abundance patterns in this study followed their feeding preferences for *M. chartacea* (Kiffer et al. 2018; Moretti et al. 2020). The lower nutritional value and the presence of secondary compounds in the leaves of *I. laurina* might have deterred a wider variety of shredder taxa (Graça and Cressa 2010). These findings further support the importance of leaf quality in shaping shredder communities.

### Leaf breakdown rates

Our results revealed that the contribution of invertebrates in leaf breakdown varied across reach types and leaf treatments. The greater mass loss of *M. chartacea* in the coarse mesh bags in both the single and mixed treatments supports the notion that shredders and other detritivores can be abundant and essential for leaf breakdown in tropical streams where high-quality leaves are available, such as in the Atlantic Forest (Casotti et al. 2019; Tonin et al. 2018). However, the relevance of invertebrates for leaf breakdown was strongly reduced in highly impacted reaches. This finding has significant ecological implications, suggesting that environmental degradation can disrupt fundamental biotic interactions and detritus-based food webs (Merz et al. 2023; Traill et al. 2010). Moreover, environmental stressors such as increased temperature, inorganic sedimentation, or pollution could further hinder detritivore activity (Carlisle and Clements 2005) or disrupt facilitative interactions between microbial decomposers and detritivores (Bernabé et al. 2018).

The lack of an overall invertebrate effect on the breakdown rates of *I. laurina* is probably related to leaf chemistry, which is rich in tannins and recalcitrant compounds. Indeed, shredders generally process leaves with high lignin: N ratios and toughness more slowly (Graça et al. 2001; Graça and Cressa 2010). Moreover, recalcitrant compounds, such as lignin, cellulose, and tannins, can hinder leaf colonization by microorganisms, reducing leaf palatability for detritivores (Graça et al. 2015; Sales et al. 2015). On the other hand, the increased leaf breakdown rates observed for *I. laurina* in the mixed treatment in the forest upstream reaches suggest a litter-mixing effect. Additive effects occur, for example, when leaves of a fast-decomposing species release nutrients (e.g., nitrogen) that can be absorbed by decomposers associated with a low-quality leaf, increasing breakdown rates (Handa et al. 2014; Merz et al. 2023). Previous research has shown that nitrogen within the microbial community can be transferred to low-quality leaves in a mixture (Hu et al. 2022). However, litter-mixing effects only emerged in environments with greater integrity, suggesting that decomposer communities in preserved reaches are functionally more effective or that mechanisms for diversity effects depend not only on functional diversity in resources but also on the decomposer community. Finally, we found no evidence that microbial-mediated leaf breakdown rates differed in open stream reaches, suggesting that higher temperatures may not necessarily enhance microbial decomposition in tropical headwater streams, or that any temperature effect was offset by other unmeasured factors. These findings underscore the complexity of leaf decomposition dynamics and highlight the importance of leaf traits and environmental conditions in shaping this process.

## Conclusions

In summary, the findings of this study—specifically, the observed improvements in water quality, increased habitat complexity, and the sustained diversity of invertebrate communities in areas influenced by forest fragments—suggest that forest fragments, both upstream and downstream of the agricultural landscape, contribute to the resilience of stream environments. This resilience is primarily supported by the role of riparian forest fragments in buffering against environmental stressors, such as increased sedimentation and temperature, through mechanisms like natural filtration and shading. Additionally, the structural complexity of riparian habitats provides refugia and resources that sustain invertebrate diversity and stabilize ecological functions, including decomposition and nutrient cycling. These results underscore the importance of protecting and restoring riparian forest fragments within mosaic catchments as a key strategy in ecosystem management (Turunen et al. 2019). From a biodiversity conservation perspective, preserving these forested areas is vital for maintaining sensitive species, such as shredders, and the ecological processes they perform, which are otherwise compromised by habitat degradation. Because riparian forests are essential for the dispersal and reproduction of adult stages of many aquatic insects (Baumgartner and Robinson 2017; Deere et al. 2022), the presence of forest fragments is also important for maintaining cross-ecosystem linkages in altered catchments. The urgency of this issue is evident in the context of the Atlantic Forest, a biome renowned for its extraordinary biodiversity, where thousands of hectares of native forest are lost yearly (Fundação SOS Mata Atlântica & INPE 2023). Therefore, immediate and targeted conservation efforts are crucial to safeguard the remaining forest fragments along stream catchments in the heavily modified but also biodiverse landscapes of the Atlantic Forest. Furthermore, future research should explore the long-term effects of riparian forest fragmentation on stream ecosystems at larger spatial scales to inform adaptive management practices in response to ongoing environmental changes.

## Supplementary Information

Below is the link to the electronic supplementary material.

Supplementary Material 1

## Data Availability

All data that support the findings of this study can be found here: https://zenodo.org/records/21949485.
