# Supplementary material for: Stream biodiversity and function benefit from forest fragments in agricultural landscapes
Source: Oecologia. 2026 Sep 24;208(11):139. doi: 10.1007/s00442-026-05977-4 (PMC13612633; doi:10.1007/s00442-026-05977-4)
Supplement: Supplementary file 1 [file 442_2026_5977_MOESM1_ESM.docx]

**Electronic Supplementary Material**

**Stream biodiversity and function benefit from forest fragments in agricultural landscapes**

Paula M. de Omena^a,b^; Larissa C. Costa^a,b^; Paula C.R. Oliveira^c^; Rebecca Oester^d,e,f^; Andreas Bruder^d^; Marcelo S. Moretti^a,b^

^a^ Laboratory of Aquatic Insect Ecology, University of Vila Velha, Av. Comissário José Dantas de Melo 21, 29102-920 Vila Velha, ES, Brazil.

^b^ Graduate Program in Plant Biotechnology, University of Vila Velha, Av. Comissário José Dantas de Melo 21, 29102-920 Vila Velha, ES, Brazil.

^c^ College of Agriculture, University of São Paulo (ESALQ), Av. Pádua Dias 11, 13418-900 Piracicaba, SP, Brazil.

^d^ Institute of Microbiology, University of Applied Sciences and Arts of Southern Switzerland, via Flora Ruchat Roncati 15, CH-6850 Mendrisio, Switzerland.

^e^ Department of Evolutionary Biology and Environmental Studies, University of Zurich, Winterthurerstr. 190, CH-8057 Zurich, Switzerland.

^f^ Eawag: Swiss Federal Institute of Aquatic Science and Technology, Department of Aquatic Ecology, Überlandstrasse 133, CH-8600 Dübendorf, Switzerland.

* Corresponding author: [paula.omena@gmail.com](mailto:paula.omena@gmail.com); phone +55 27 996619840

ORCIDs:
PMO: 0000-0002-5221-7901

LCC: 0000-0002-2821-2561

PCRO: 0000-0001-7451-3659

RO: 0000-0002-9192-6882

AB: 0000-0002-4591-491X

MSM:0000-0003-1994-2105

**Table S1** Environmental characteristics and water parameters (mean ± SD) measured at 16 stream reaches in catchments altered by land use. The reach types include forests upstream, forests downstream, deforested downstream, and deforested upstream.

| **Environmental characteristics** | **Forest upstream** | **Forest downstream** | **Deforested downstream** | **Deforested upstream** |
| --- | --- | --- | --- | --- |
| Canopy cover (%) | 62.4 ± 2.49 | 61.6 ± 3.10 | 28.6 ± 27.5 | 27 ± 17.7 |
| Stream width (m) | 1.39 ± 0.40 | 1.68 ± 0.54 | 1.24 ± 0.45 | 1.3 ± 0.70 |
| Stream depth (cm) | 15.6 ± 7.32 | 18.6 ± 3.65 | 15.6 ± 4.25 | 17 ± 9.34 |
| Water velocity (m.s^-1^) | 0.17 ± 0.06 | 0.2 ± 0.06 | 0.14 ± 0.05 | 0.23 ± 0.03 |
| Canopy cover (%) | 62.4 ± 2.49 | 61.6 ± 3.1 | 28.6 ± 27.52 | 27 ± 17.7 |
| Forest cover (%)* | 52 ± 9.48 | 77.7 ± 17.9 | 12.8 ± 23 | 24.7 ± 22 |
| Daily mean temperature (°C) | 20 ± 1.82 | 21.6 ± 2.00 | 20.2 ± 1.89 | 22 ± 2.04 |
| Daily mean temperature oscillation (°C) | 1.48 ± 0.49 | 2.69 ± 0.89 | 1.94 ± 0.2 | 2.83 ± 1.71 |
| pH | 6.15 ± 0.49 | 6 ± 0.34 | 6.18 ± 0.38 | 5.74 ± 0.53 |
| Electrical conductivity (μS.cm^-1^) | 0.03 ± 0.004 | 0.041 ± 0.007 | 0.027 ± 0.009 | 0.04 ± 0.013 |
| Dissolved oxygen (mg.L^-1^) | 9.61 ± 2.16 | 9.44 ± 2.08 | 9.43 ± 1.59 | 8.75 ± 1.73 |
| Redox potential (mV) | 199.1 ± 68.5 | 228 ± 33.8 | 204 ± 62.8 | 231.3 ± 37.1 |
| Nitrate (mg.L^-1^) | 0.66 ± 0.51 | 0.85 ± 0.59 | 0.61 ± 0.48 | 0.97 ± 0.71 |
| Organic sedimentation rate (mg.d^-1^) | 17.5 ± 10.2 | 12.1 ± 8.12 | 18.8 ± 12.7 | 12 ± 8.75 |
| Inorganic sedimentation rate (mg.d^-1^) | 45.5 ± 14.6 | 60.4 ± 8.68 | 49.9 ± 10.5 | 61.5 ± 9.74 |
| Substrate diversity (H') | 1.56 ± 0.07 | 1.44 ± 0.40 | 1.30 ± 0.36 | 1.28 ± 0.33 |
| Leaf patches (%) | 21.2 ± 15.1 | 11.4 ± 11.9 | 8.5 ± 9.05 | 3.2 ± 5.19 |

* buffer radius 50 m

**Table S2** Summary of the principal component analysis of *Inga laurina* and *Miconia chartacea* leaf attributes and of each leaf attribute's mean value and standard deviation (SD). Leaf attributes that have significant correlations with the PC axis are shown in bold.

| **Leaf attributes** | **PC1** | **PC2** | ***I. laurina***  **(mean ± SD)** | | ***M. chartacea***  **(mean ± SD)** |
| --- | --- | --- | --- | --- | --- |
| C | 0.31 | **0.91** | 39.68 ± 1.76 | 38.50 ± 2.32 | |
| N | **-0.99** | 0.04 | 1.30 ± 0.02 | 1.75 ± 0.10 | |
| P | **-0.82** | 0.44 | 0.02 ± 0.003 | 0.04 ± 0.01 | |
| K | **-0.98** | 0.13 | 0.10 ± 0.02 | 0.60 ± 0.13 | |
| C:N | **0.96** | 0.24 | 30.40 ± 1.87 | 22.04 ± 1.60 | |
| Lignin:N | **0.98** | 0.06 | 38.24 ± 1.62 | 17.59 ± 1.16 | |
| Tannin | **0.94** | 0.03 | 3.81 ± 0.036 | 0.88 ± 0.06 | |
| Cellulose | **0.88** | -0.19 | 38.71 ± 0.75 | 34.77 ± 2.53 | |
| Fibers | **-0.99** | -0.02 | 6.30 ± 1.56 | 28.40 ± 2.64 | |
| Toughness | **0.97** | 0.07 | 452.73 ± 32.78 | 275.06 ± 14.13 | |
| *Variance explained* | 82.7% | 11.7% |  |  | |

**Table S3** Summary of the principal component analysis of the stream environmental characteristics and water parameters. The environmental characteristics and water parameters that have significant correlations with the PC axis are shown in bold.

| **Environmental characteristic** | **PC1** | **PC2** |
| --- | --- | --- |
| Electric conductivity (μS/cm) | -0.59 | **0.68** |
| pH | **0.74** | -0.22 |
| Substrate diversity (H') | **0.74** | 0.16 |
| Inorganic sedimentation rate (mg/d) | **-0.66** | 0.08 |
| Water velocity (ms^-1^) | -0.18 | **0.55** |
| Temperature oscillation (°C) | **-0.73** | 0.19 |
| Canopy cover (%) | 0.56 | **0.68** |
| Leaf patches (%) | **0.82** | 0.14 |
| Forest cover (%) | 0.26 | **0.90** |
| *Variance explained* | 39.6% | 24.7% |

**Table S4** Model forms, effects, and results for all GLMMs used to investigate the effects of reach type (R) and leaf treatment (L) on the abundance of aquatic invertebrate assemblages. Bold text indicates significance at p = 0.05. All of the models included stream as a random effect. The intercept for the reach type in the models is R. F up (i.e., forest upstream), and the intercept for the leaf treatment is L. Miconia (*Miconia chartacea* single). The codes for the fixed effects are R. F down = forest downstream, R. D down = deforested downstream, R. D up = deforested upstream, L. Inga = *Inga laurina* single, L. Mixed = mixed leaf treatment.

| **Response Variable** | **Model** | **Model family/link** | **Fixed Effect** | **Estimates** | **SE** | ***Z*** | ***p*** | **R^2^_m_** | **R^2^_c_** |
| --- | --- | --- | --- | --- | --- | --- | --- | --- | --- |
| Shredders | R + L | Tweedie  (log link) | Intercept | 0.96 | 0.33 | 2.85 | 0.004 | 9.3% | 70% |
|  |  |  | R. F down | -0.10 | 0.46 | -0.21 | 0.83 |  |  |
|  |  |  | R. D down | -0.16 | 0.15 | -1.1 | 0.27 |  |  |
|  |  |  | R. D up | -0.38 | 0.47 | -0.81 | 0.41 |  |  |
|  |  |  | L. Inga | -0.46 | 0.14 | -3.37 | **<0.001** |  |  |
|  |  |  | L. Mixed | -0.15 | 0.13 | -1.17 | 0.24 |  |  |
| Collector-gatherers | R + L | Neg. binomial  (log link) | Intercept | 3.44 | 0.43 | 7.84 | <0.0001 | 12.3% | 60.9% |
|  |  |  | R. F down | 0.38 | 0.61 | 0.62 | 0.53 |  |  |
|  |  |  | R. D down | 0.36 | 0.19 | 1.92 | **0.05** |  |  |
|  |  |  | R. D up | 1.12 | 0.60 | 1.85 | **0.06^*^** |  |  |
|  |  |  | L. Inga | -0.03 | 0.16 | -0.19 | 0.84 |  |  |
|  |  |  | L. Mixed | 0.05 | 0.16 | 0.34 | 0.72 |  |  |
| Collector-filterers | R + L | Tweedie  (log link) | Intercept | 0.90 | 0.65 | 1.39 | 0.16 | 49.7% | 80% |
|  |  |  | R. F down | 1.94 | 0.88 | 2.19 | **0.02** |  |  |
|  |  |  | R. D down | -0.17 | 0.33 | -0.50 | 0.61 |  |  |
|  |  |  | R. D up | 3.45 | 0.88 | 3.92 | **<0.0001** |  |  |
|  |  |  | L. Inga | -0.02 | 0.21 | -0.12 | 0.90 |  |  |
|  |  |  | L. Mixed | 0.23 | 0.21 | 1.10 | 0.27 |  |  |
| Scrapers | R + L | Tweedie  (log link) | Intercept | 1.24 | 0.75 | 1.64 | 0.09 | 7.3% | 72.9% |
|  |  |  | R. F down | 0.19 | 1.05 | 0.18 | 0.85 |  |  |
|  |  |  | R. D down | 0.92 | 0.22 | 4.09 | **<0.0001** |  |  |
|  |  |  | R. D up | 1.14 | 1.05 | 1.08 | 0.27 |  |  |
|  |  |  | L. Inga | 0.007 | 0.19 | 0.03 | 0.96 |  |  |
|  |  |  | L. Mixed | -0.04 | 0.20 | -0.24 | 0.81 |  |  |
| Predators | R + L | Neg. binomial  (log link) | Intercept | 2.21 | 0.38 | 5.80 | <0.0001 | 12.4% | 57.1% |
|  |  |  | R. F down | 0.94 | 0.52 | 1.8 | 0.07* |  |  |
|  |  |  | R. D down | 0.66 | 0.17 | 3.88 | **0.0001** |  |  |
|  |  |  | R. D up | 0.82 | 0.52 | 1.56 | 0.11 |  |  |
|  |  |  | L. Inga | -0.10 | 0.14 | -0.71 | 0.47 |  |  |
|  |  |  | L. Mixed | 0.001 | 0.15 | 0.01 | 0.99 |  |  |
| EPT | R + L | Neg. binomial  (log link) | Intercept | 2.48 | 0.58 | 4.23 | <0.0001 | 10% | 63.9% |
|  |  |  | R. F down | 0.14 | 0.81 | 0.17 | 0.85 |  |  |
|  |  |  | R. D down | 0.17 | 0.25 | 0.71 | 0.47 |  |  |
|  |  |  | R. D up | 1.18 | 0.81 | 1.45 | 0.14 |  |  |
|  |  |  | L. Inga | 0.06 | 0.21 | 0.29 | 0.77 |  |  |
|  |  |  | L. Mixed | -0.02 | 0.21 | -0.10 | 0.91 |  |  |

**Table S5** Mean and standard deviation values of the abundance and diversity (H') of invertebrate functional feeding groups and EPT taxa in the different stream reach types (forest upstream, forest downstream, deforested upstream, and deforested downstream).

| **Invertebrates** | **Forest**  **upstream** | **Forest downstream** | **Deforested downstream** | **Deforested upstream** |
| --- | --- | --- | --- | --- |
| *Abundance* |  |  |  |  |
| Shredders | 6.34 ± 5.67 | 11 ± 21.65 | 5.58 ± 7.01 | 7 ± 20.57 |
| Collector-gatherers | 51 ± 65.97 | 49 ± 58.51 | 83 ± 111.21 | 97.61 ± 14 |
| Collector-filterers | 3 ± 5.04 | 33 ± 71.32 | 3 ± 4.48 | 173 ± 256.31 |
| Scrapers | 8 ± 12.97 | 4 ± 7.09 | 28 ± 48.65 | 14 ± 25.71 |
| Predators | 13 ± 23.66 | 20.85 ± 13.80 | 23.54 ± 21.96 | 22 ± 40.39 |
| EPT | 16 ± 20.31 | 16 ± 25.51 | 36 ± 54.88 | 60 ± 154.08 |
|  |  |  |  |  |
| *Diversity H'* |  |  |  |  |
| Shredders | 0.41 ± 0.42 | 0.28 ± 0.36 | 0.40 ± 0.05 | 0.18 ± 0.02 |
| Collector-gatherers | 0.91 ± 0.48 | 1 ± 0.32 | 1.09 ± 0.42 | 0.82 ± 0.47 |
| Collector-filterers | 0.08 ± 0.23 | 0.15 ± 0.27 | 0.13 ± 0.28 | 0.27 ± 0.31 |
| Scrapers | 0.33 ± 0.40 | 0.19 ± 0.30 | 0.50 ± 0.61 | 0.47 ± 0.44 |
| Predators | 0.81 ± 0.53 | 1.14 ± 0.35 | 0.86 ± 0.37 | 1.04 ± 0.59 |
| EPT | 0.86 ± 0.52 | 0.59 ± 0.42 | 0.98 ± 0.68 | 0.55 ± 0.08 |

**Table S6** Model forms, effects, and results for all GLMMs used to investigate the effects of reach type (R) and leaf treatment (L) on the diversity of aquatic invertebrate assemblages. The description of the table is the same as that of Table S4. For the models with a Gaussian distribution, the values for the statistical test are t, and for the Tweedie distribution, the values are z. All of the models included stream as a random effect.

| **Response Variable** | **Model** | **Model family/link** | **Fixed Effect** | **Estimates** | **SD** | ***Z* or *t*** | ***p*** | **R^2^_m_** | **R^2^_c_** |
| --- | --- | --- | --- | --- | --- | --- | --- | --- | --- |
| Shredders | R + L | Tweedie  (log link) | Intercept | -0.79 | 0.42 | -1.88 | 0.05 | 23.3% | 45.3% |
|  |  |  | R.F down | -0.50 | 0.59 | -0.84 | 0.39 |  |  |
|  |  |  | R.D down | -0.19 | 0.26 | -0.76 | 0.44 |  |  |
|  |  |  | R.D up | -1.77 | 0.68 | -2.57 | **0.009** |  |  |
|  |  |  | L. Inga | -0.52 | 0.27 | -1.94 | 0.05* |  |  |
|  |  |  | L. Mixed | -0.09 | 0.25 | -0.34 | 0.72 |  |  |
| Collector-gatherers | R + L | Gaussian  (identity) | Intercept | 0.96 | 0.15 | 6.41 | <0.0001 | 4% | 38% |
|  |  |  | R.F down | 0.06 | 0.20 | 0.31 | 0.75 |  |  |
|  |  |  | R.D down | 0.15 | 0.07 | 2.01 | **0.04** |  |  |
|  |  |  | S.D up | -0.08 | 0.20 | -0.43 | 0.66 |  |  |
|  |  |  | L. Inga | -0.06 | 0.06 | -0.96 | 0.33 |  |  |
|  |  |  | L. Mixed | -0.02 | 0.07 | -0.35 | 0.72 |  |  |
| Collector-filterers | R + L | Tweedie  (log link) | Intercept | -1.89 | 0.49 | -3.87 | 0.0001 | 3% | 32.6% |
|  |  |  | R.F down | 0.19 | 0.64 | 0.31 | 0.76 |  |  |
|  |  |  | R.D down | 0.22 | 0.34 | 0.63 | 0.52 |  |  |
|  |  |  | R.D up | 0.67 | 0.63 | 1.06 | 0.28 |  |  |
|  |  |  | L. Inga | -0.26 | 0.30 | -0.85 | 0.39 |  |  |
|  |  |  | L. Mixed | 0.004 | 0.29 | 0.01 | 0.98 |  |  |
| Scrapers | R + L | Tweedie  (log link) | Intercept | -2.02 | 1.05 | -1.92 | 0.05 | 2% | 77.6% |
|  |  |  | R.F down | -0.39 | 1.46 | -0.27 | 0.78 |  |  |
|  |  |  | R.D down | 0.31 | 0.20 | 1.54 | 0.12 |  |  |
|  |  |  | R.D up | 0.47 | 1.45 | 0.32 | 0.74 |  |  |
|  |  |  | L. Inga | -0.12 | 0.18 | -0.66 | 0.51 |  |  |
|  |  |  | L. Mixed | 0.11 | 0.17 | 0.64 | 0.52 |  |  |
| Predators | R + L | Gaussian  (identity) | Intercept | 0.82 | 0.14 | 5.76 | <0.0001 | 9.5% | 24.3% |
|  |  |  | R.F down | 0.33 | 0.17 | 1.88 | **0.04** |  |  |
|  |  |  | R.D down | 0.04 | 0.09 | 0.44 | 0.65 |  |  |
|  |  |  | R.D up | 0.29 | 0.17 | 1.66 | 0.11 |  |  |
|  |  |  | L. Inga | -0.07 | 0.08 | -0.94 | 0.47 |  |  |
|  |  |  | L. Mixed | 0.07 | 0.08 | 0.89 | 0.33 |  |  |
| EPT | R + L | Gaussian  (identity) | Intercept | 0.91 | 0.21 | 4.20 | <0.001 | 8% | 51.6% |
|  |  |  | R.F down | -0.29 | 0.29 | -0.97 | 0.33 |  |  |
|  |  |  | R.D down | 0.08 | 0.09 | 0.911 | 0.36 |  |  |
|  |  |  | R.D up | -0.32 | 0.30 | -1.09 | 0.27 |  |  |
|  |  |  | L. Inga | -0.04 | 0.07 | -0.56 | 0.57 |  |  |
|  |  |  | L. Mixed | 0.03 | 0.08 | 0.40 | 0.68 |  |  |

**Table S7** Model forms, effects, and results for all GLMMs used to investigate the effects of reach type (R) and the presence of invertebrates (mesh size: MS) on the leaf breakdown of *Miconia chartacea* and *Inga laurina* in both the single- and mixed-leaf treatments. In these models, the intercepts MS represent the coarse mesh size, and MS.Fine represents the fine mesh size. We square-root transformed the response variable in all of the models to control for overdispersion. Additionally, all of the models included the stream as a random effect. For all of the models, the family distribution was Gaussian with a link function identity.

| **Response Variable** | **Model** | **Fixed Effect** | **Estimates** | **SD** | ***t*** | ***p*** | **R^2^_m_** | **R^2^_c_** |
| --- | --- | --- | --- | --- | --- | --- | --- | --- |
| K *_Miconia_* _single_ | R + MS | Intercept | 0.032 | 0.002 | 15.71 | <0.001 | 0.36 | 0.64 |
|  |  | R.F down | -0.004 | 0.003 | -1.41 | 0.162 |  |  |
|  |  | R.D down | -0.003 | 0.001 | -2.33 | **0.022** |  |  |
|  |  | R.D up | -0.01 | 0.003 | -3.54 | **0.001** |  |  |
|  |  | MS.Fine | -0.009 | 0.001 | -6.65 | **<0.001** |  |  |
|  |  | R.F down* MS.Fine | 0.003 | 0.00 | 1.67 | 0.097 |  |  |
|  |  | R.Ddown* MS.Fine | 0.002 | 0.001 | 1.12 | 0.262 |  |  |
|  |  | R.D up* MS.Fine | 0.008 | 0.002 | 4.28 | **<0.001** |  |  |
| K *_Miconia_* _mixed_ | R + MS | Intercept | 0.028 | 0.001 | 14.98 | <0.001 | 0.17 | 0.41 |
|  |  | R.F down | -0.0006 | 0.002 | -0.25 | 0.798 |  |  |
|  |  | R.D down | -0.003 | 0.001 | -1.73 | 0.086 |  |  |
|  |  | R.D up | -0.004 | 0.002 | -1.73 | 0.086 |  |  |
|  |  | MS.Fine | -0.006 | 0.001 | -4.11 | **<0.001** |  |  |
|  |  | R.Fdown* MS.Fine | 0.001 | 0.002 | 0.65 | 0.514 |  |  |
|  |  | R.Ddown* MS.Fine | 0.004 | 0.002 | 1.79 | **0.075** |  |  |
|  |  | R.Dup* MS.Fine | 0.006 | 0.002 | 2.55 | **0.012** |  |  |
| K *_Inga_* _single_ | R + MS | Intercept | 0.015 | 0.0005 | 26.6 | <0.001 | 0.13 | 0.37 |
|  |  | R.F down | -0.001 | 0.0008 | -2.15 | **0.03** |  |  |
|  |  | R.D down | -0.001 | 0.0005 | -1.86 | 0.06 |  |  |
|  |  | R.D up | -0.002 | 0.0008 | -2.56 | **0.01** |  |  |
|  |  | MS.Fine | -0.001 | 0.0005 | -1.98 | 0.05 |  |  |
|  |  | R.F down* MS.Fine | 0.001 | 0.0007 | 1.19 | 0.233 |  |  |
|  |  | R.Ddown* MS.Fine | 0.001 | 0.0007 | 1.56 | 0.12 |  |  |
|  |  | R.Dup* MS.Fine | 0.001 | 0.0007 | 1.59 | 0.11 |  |  |
| K *_Inga_* _mixed_ | R + MS | Intercept | 0.015 | 0.0007 | 20.80 | **0.01** | 0.11 | 0.34 |
|  |  | R.F down | -0.002 | 0.001 | -2.45 | **0.01** |  |  |
|  |  | R.D down | -0.001 | 0.0007 | -2.57 | **0.01** |  |  |
|  |  | R.D up | -0.002 | 0.001 | -2.31 | **0.02** |  |  |
|  |  | MS.Fine | -0.001 | 0.0007 | -1.92 | 0.05 |  |  |
|  |  | R.F down* MS.Fine | 0.002 | 0.001 | 2.16 | **0.03** |  |  |
|  |  | R.Ddown* MS.Fine | 0.001 | 0.001 | 1.41 | 0.16 |  |  |
|  |  | R.Dup* MS.Fine | 0.001 | 0.001 | 1.07 | 0.28 |  |  |

**Table S8** Mean and standard deviation values of the leaf breakdown rates (*k*) and percentage of leaf mass lost from the leaves of *Miconia chartacea* and *Inga laurina* by stream reach condition (forest upstream, forest downstream, deforested upstream, deforested downstream), leaf treatment (single and mixed), and mesh size (coarse and fine).

| **Reach type** | **Leaf** | **Mesh size** | ***k* (mg dd^-1^)** | **SD** | **ML (%)** | **SD** |
| --- | --- | --- | --- | --- | --- | --- |
| Forest  upstream | *Miconia* | Coarse | 0.00107 | 0.000437 | 61.01 | 17.65 |
|  |  | Fine | 0.00053 | 0.000183 | 38.75 | 11.80 |
|  | *Miconia*  mixed | Coarse | 0.00081 | 0.000538 | 48.28 | 15.89 |
|  |  | Fine | 0.00047 | 0.000190 | 35.36 | 13.78 |
|  | *Inga* | Coarse | 0.00024 | 0.000116 | 19.78 | 9.18 |
|  |  | Fine | 0.00020 | 0.000019 | 17.17 | 2.67 |
|  | *Inga*  mixed | Coarse | 0.00028 | 0.000238 | 21.73 | 13.33 |
|  |  | Fine | 0.00021 | 0.000026 | 18.21 | 2.067 |
| Forest  downstream | *Miconia* | Coarse | 0.00079 | 0.000305 | 52.53 | 13.93 |
|  |  | Fine | 0.00049 | 0.000095 | 38.26 | 6.625 |
|  | *Miconia*  mixed | Coarse | 0.00078 | 0.000358 | 50.82 | 16.25 |
|  |  | Fine | 0.00050 | 0.000134 | 38.56 | 9.08 |
|  | *Inga* | Coarse | 0.00018 | 0.000039 | 16.03 | 3.45 |
|  |  | Fine | 0.00018 | 0.000031 | 15.94 | 2.89 |
|  | *Inga*  mixed | Coarse | 0.00018 | 0.000024 | 16.13 | 2.32 |
|  |  | Fine | 0.00021 | 0.000075 | 18.14 | 6.07 |
| Deforested  upstream | *Miconia* | Coarse | 0.00047 | 0.00026 | 35.11 | 12.97 |
|  |  | Fine | 0.00046 | 0.000156 | 36.38 | 8.83 |
|  | *Miconia*  mixed | Coarse | 0.00054 | 0.000161 | 40.49 | 9.13 |
|  |  | Fine | 0.00052 | 0.000162 | 40.02 | 8.07 |
|  | *Inga* | Coarse | 0.00017 | 0.000037 | 15.84 | 3.33 |
|  |  | Fine | 0.00018 | 0.000043 | 16.14 | 3.62 |
|  | *Inga*  mixed | Coarse | 0.00018 | 0.000049 | 16.81 | 4.19 |
|  |  | Fine | 0.00018 | 0.000035 | 16.05 | 2.55 |
| Deforested downstream | *Miconia* | Coarse | 0.00089 | 0.000470 | 53.08 | 14.46 |
|  |  | Fine | 0.00048 | 0.000110 | 36.04 | 8.23 |
|  | *Miconia*  mixed | Coarse | 0.00066 | 0.000262 | 44.72 | 13.69 |
|  |  | Fine | 0.00052 | 0.000202 | 37.64 | 9.96 |
|  | *Inga* | Coarse | 0.00020 | 0.000034 | 17.27 | 3.05 |
|  |  | Fine | 0.00020 | 0.000036 | 17.45 | 3.78 |
|  | *Inga*  mixed | Coarse | 0.00020 | 0.000033 | 17.30 | 3.22 |
|  |  | Fine | 0.00020 | 0.000032 | 17.44 | 3.30 |

**Fig. S1** Mean forest cover proportion (± SD) at sampling points within buffer zones of 30, 50, and 100 meters in radius. Data are separated by riparian vegetation type, with "Deforested" (●) and "Forest" (▲) categories

**Fig. S2** Principal component analysis (PCA) summarizing the correlations between the nine leaf attributes measured for leaves of *Inga laurina* and *Miconia chartacea*, which were randomly chosen from the initial pool of leaves before the start of the experiments. We only show leaf attributes with correlations of r > 0.5 and p < 0.05

**Leaf-associated inverterbrates**

A total of 28,465 invertebrates associated with the leaves, representing 135 taxa, were identified primarily at the genus and family levels (Fig. S3). Ostracoda was the most abundant taxon (5,879 ind., 20.6%), followed by Tanytarsini (Chironomidae; 5,276 ind., 18.5%), *Smicridea* sp*.* (Hydropsychidae; 2,003 ind., 7.0%), Oligochaeta (1,933 ind., 6.8%), Tanypodinae (Chironomidae; 1,789 ind., 6.3%), and Orthocladiinae (Chironomidae; 1,631 ind., 5.7%). The collector-gatherers were the most abundant functional feeding group (12,174 ind.), followed by the collector-filters (8,922 ind.), scrapers (2,491 ind.), predators (3,493 ind.) and shredders (1,339 ind.). Eleven taxa of shredders were identified, with the most abundant being *Heterelmis* sp. (Elmidae; 809 ind.), *Stenochironomus* sp. (Chironomidae; 263 ind.), *Phylloicus* sp. (Calamoceratidae; 134 ind.), *Oecetis* sp. (Leptoceridae; 94 ind.), and *Triplectides* sp. (Leptoceridae; 60 ind.).

**Fig. S3** The proportional abundances of the main taxa (n > 10 ind.) of leaf-associated invertebrates found in streams under different environmental conditions as follows: forest upstream (dark green), forest downstream (light green), deforested downstream (light purple), and deforested upstream (dark purple). The taxa are ordered according to their total abundance, from the most abundant (Ostracoda, n = 5,879 ind.) to the least abundant (Ancylidae, Leptophlebiidae, *Macronema*, and *Progomphus*, n = 11 ind.). The functional feeding groups are indicated by the following codes: collector-filterers (Cf), collector-gatherers (Cg), scrapers (Sc), shredders (Sh), predators (P), and not assigned (na)

**Fig. S4** Abundances of shredders (mean ± SD) associated with leaves of *Miconia chartacea* and *Inga laurina* in the single and mixed treatments
